# Revisiting the role of mesenchymal stromal cells in cancer initiation, metastasis and immunosuppression

**DOI:** 10.1186/s40164-024-00532-4

**Published:** 2024-07-01

**Authors:** Yanyan Zhang, Charles Wang, Jian Jian Li

**Affiliations:** 1 Jiangsu Cancer Hospital, Jiangsu Institute of Cancer Research, The Affiliated Cancer Hospital of Nanjing Medical University, Nanjing, Jiangsu China; 2 Department of Radiation Oncology, School of Medicine, University of California Davis, Sacramento, CA USA; 3 NCI-Designated Comprehensive Cancer Center, University of California Davis, Sacramento, CA 95817 USA

**Keywords:** Immunotherapy, Mesenchymal stromal cells, Tumor-imitating cells, Tumor microenvironment, Tumor-associated MSCs, Immunosuppression, Radiotherapy

## Abstract

Immune checkpoint blockade (ICB) necessitates a thorough understanding of intricate cellular interactions within the tumor microenvironment (TME). Mesenchymal stromal cells (MSCs) play a pivotal role in cancer generation, progression, and immunosuppressive tumor microenvironment. Within the TME, MSCs encompass both resident and circulating counterparts that dynamically communicate and actively participate in TME immunosurveillance and response to ICB. This review aims to reevaluate various facets of MSCs, including their potential self-transformation to function as cancer-initiating cells and contributions to the creation of a conducive environment for tumor proliferation and metastasis. Additionally, we explore the immune regulatory functions of tumor-associated MSCs (TA-MSCs) and MSC-derived extracellular vesicles (MSC-EVs) with analysis of potential connections between circulating and tissue-resident MSCs. A comprehensive understanding of the dynamics of MSC-immune cell communication and the heterogeneous cargo of tumor-educated versus naïve MSCs may unveil a new MSC-mediated immunosuppressive pathway that can be targeted to enhance cancer control by ICB.

## Background

Multiple intrinsic cascades of genomic and epigenetic events denoted as the hallmarks of cancer are believed to drive the malignant cell transformation and tumor progression [1, 2]. However, in addition to the well-defined cell intrinsic pre-cancer trends, increasing evidence indicates a potential systemic and/or tissue environmental factor leading to a global pre-cancerous status [3–5]. Such a pre-cancerous environmental status is triggered by imbalanced tissue homeostasis and architecture far before the malignant phenotype can be noticed [6]. Consistently, non-mutational epigenetic events and senescent cells are suggested to play an effective role in the tumor microenvironment (TME) [3], and cancer origination, aggressiveness, and metastasis are indicated to be the result of communications between pre-cancer cells or tumors and the extracellular environment [7]. This central dogma of cancer is evidenced by the well-defined pro-cancer chronic inflammation that generates pro-inflammatory cytokines and growth factors [7–9]. It is assumed that even at the very early tumor initiation phase, a variety of cell types from local and distant niches are recruited into the growing neoplasm to provide stromal support for the transformed cells [10]. Such a pre-cancerous stroma can further attract different regulatory molecules to mobilize an array of cell types to join in the "transforming niche," in which mesenchymal stromal cells (MSCs) may guide the process.

In adult humans, MSCs are generally identified by detecting several non-hematopoietic markers, e.g., CD29, CD44, CD73, CD90, CD105, and by the characteristics of differentiating towards diverse cell types, including adipocytes, osteoblasts, chondrocytes, and connective tissues [11]. In addition to the homing function of circulating MSCs, a vast number of tissue-resident MSCs are primarily located in perivascular sites and are capable of quickly responding to external stimuli to differentiate into pericytes to regulate vascular morphogenesis and biological functions [12, 13]. Due to their injury-homing properties, along with the relative enriched resources for isolation and in vitro expansion, priming MSCs or engineered MSC/MSC-EVs are extensively investigated for disease treatment [14], and regenerative and drug delivery therapies [15]. MSCs-regulated immune inhibition offers a desirable approach in adjuvant immunomodulation post-allogeneic transplantation [16, 17] and cancer control [18]. Companying with the wholesale clinical applications, MSCs from different tissue resources are cautioned by a potential risk of malignant initiation and cancer progression owning to their stem cell features and immunosuppressive potential. The tissue-resident MSCs include the TA-MSCs highlighted to be a critical element in TME [18, 19], and targeting TA-MSCs by different approaches including radiotherapy diminished tumor aggressiveness [20]. Given that myriad factors influence the phenotype and function of MSCs within the TME [21], it holds paramount significance to further elucidate the fundamental feature of MSCs beyond the communication and coordination with tumor and immune cells in the TME.

## MSCs as the potential cancer-initiating cells

### Animal MSCs self-transformation

**Fig. 1:**
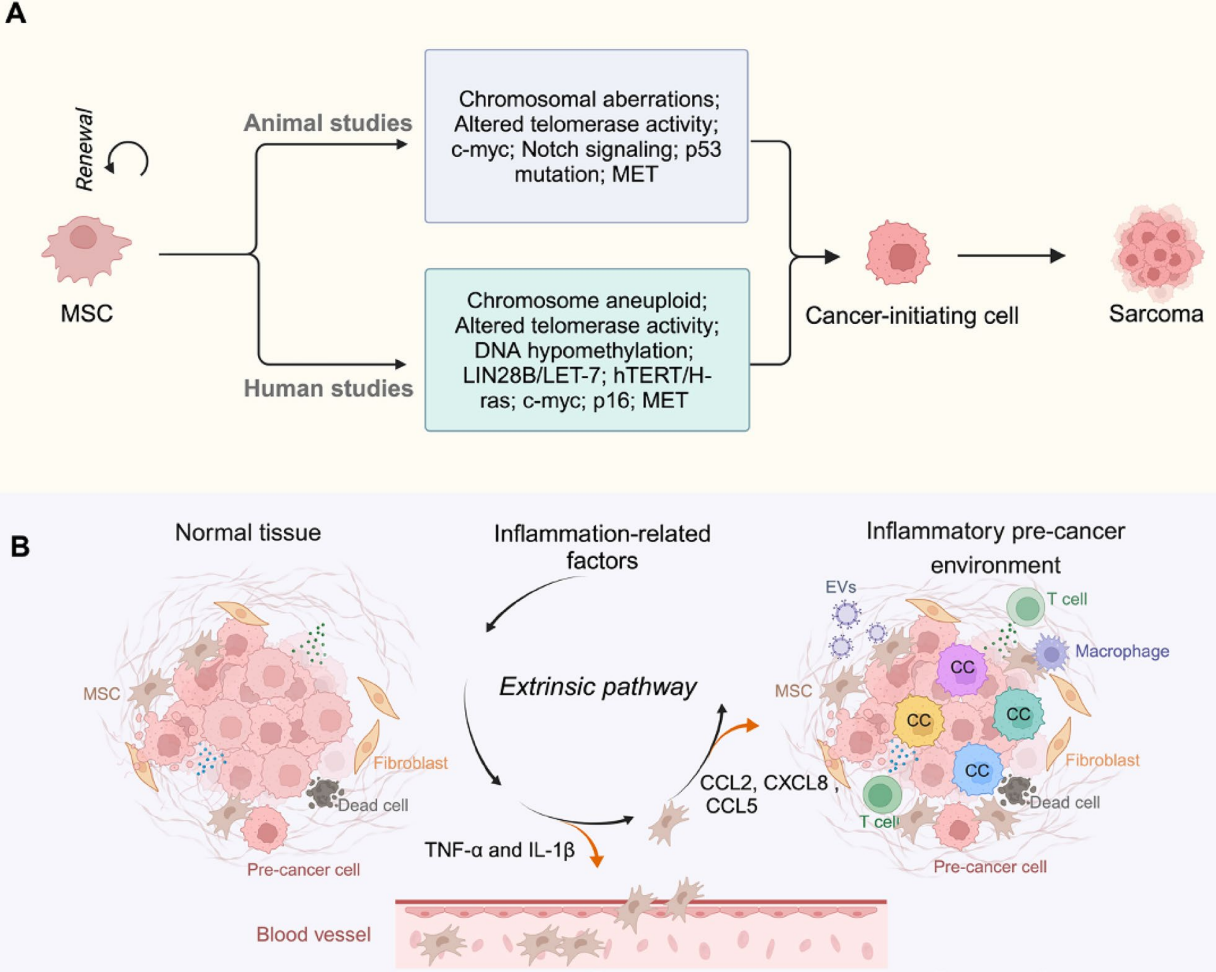
MSCs serve as cancer-initiating cells via self-transformation and/or pro-cancerous niches. **A** In animal models, MSCs can spontaneously transform vitro and in vivo (sarcoma formation) by cytogenetic abnormalities, chromosomal aberrations, telomerase activity alterations, c-myc expression, p53 mutation, MET and Notch( +)/Hh(−)/Wnt(−) signaling pathway. In human, MSCs self-transformation depends on chromosome aneuploid, altered telomerase activity, LIN28B/LET-7, hTERT/H-ras, c-myc, p16, DNA hypomethylation, MET. **B** Different genotoxic and cellular stress conditions including UV, ionizing radiation, virus infection, and chemical carcinogens, lead to a pro-cancer niche with the recruitment of inflammatory cells, and the resident and/or circulating MSCs. Damages or loss of homeostasis of the local epithelial tissue enhance the homing of MSCs followed by attraction of an array of the inflammatory cells and their derived cytokines that triggers the oncogenic transformation of stem cell or progenitor cells initiating a malignant proliferative cancer niche in which the precise cellular resource of the pre-tumor cells remains unelucidated (cc, cancer cells)

Observations that lower organisms can regenerate multiple tissues and organs have led to the proposal of somatic stem cells (SCs) [22], which are generally believed to be a small fraction of somatic cells capable of self-renewing and are responsible for tissue regeneration and homeostasis [23, 24]. Experimental data suggest that SCs share some essential features with the clonogenic tumor cells or the tumor-initiating cells (TICs) illustrating the similarity between the two types of cells [25]. With this hypothesis, MSCs are assumed to be the original cellular resources holding the pre-cancerous trends when a certain level of cancer hallmark gene mutation is achieved through accumulated cell division times [26, 27]. Additionally, it has been identified that a single passage in culture extensively alters MSCs molecular signatures associated with cell cycling, differentiation, and immune response, highlighting the need to clarify the consequence of the transition [28]. However, unlike fully transformed cancer cells, MSCs demonstrate both pro- and anti-tumor functions [29], thus, the precise biological connections between MSCs and TICs remain to be further elucidated. Although vast evidence illustrates that mouse bone marrow-derived mesenchymal stromal cells (mBMSCs) promote tumor growth in cancer-carrying mice, the confidence level of true transformation of MSCs in vivo is still under debate, and DNA fingerprinting is suggested in such experimental settings [30–33]. Nonetheless, the following reports are cited supporting the concept that mMSCs can spontaneously undergo transformation in vitro and generate sarcoma in vivo [34, 35] (Fig. 1A). Using the tracing approach in aged mice, Houghton’s group demonstrates that MSCs were able to spontaneously transform with p53 mutation leading to fibrosarcoma generation. Of note, the transformed MSCs not only rooted the tumor mass but also generated the tumor-associated vasculature and stromal supports [36]. Similarly, CD44^+^/CD29^+^ mBMSCs transplanted into myocardial infarction and diabetic neuropathy mice resulted in the formation of malignant sarcoma with chromosomal aberrations including fusion, fragmentation, and ring formation [37]. mBMSCs can also acquire immortality with transformation behavior and fibrosarcoma formation via altered telomerase activity, c-myc expression [38], and Notch^+^/Hh^−^/Wnt^−^ signaling pathway[39]. In addition, MSCs derived from cynomolgus monkeys were tumor-generative after long-term in vitro expansion [40]. Such pro-transformative tendencies in MSCs could be unique, as they are not observed in most other mouse stem cells, including hematopoietic and embryonic stem cells [41]. A recent report further demonstrated that with single-cell RNAseq and lineage tracing approaches mMSCs can undergo mesenchymal-epithelial transition (MET) and be incorporated into the re-epithelialized luminal surface of the repaired tissue [42]. However, it remains to be further characterized and verified how cancer cells could at least in part result from a potential intermediate cell type derived from mMSCs via MET.

### Human MSCs self-transformation

Similarly, to animal models, the idea of human MSCs (hMSCs) being the original source of both epithelial and non-epithelial malignancy is still a topic of debate. Long-term in vitro expansion of hMSCs can induce senescence-associated phenotype with chromosomal alterations including aneuploidy and polyploidy but no self-transformation is detected [43, 44]. However, variable aneuploid clone proportions were identified in a large group of hMSCs suggesting a potential transform trend [45]. A pioneer work by Wang’s group provided the first in vivo evidence that BMSCs are the potential cancer originating cells in a mouse gastric tumor model [46]. Such evidence has been further strengthened by a series of studies supporting that the spontaneous transformation of hBMSCs can be enhanced by LIN28B expression leading to sarcoma formation in immunocompromised mice, suggesting a prognostic factor for clinic sarcoma patients [47]. The potential hMSCs tumorigenic potential was achieved as high as 45.8% by in vitro expansion [48] with a significantly proliferative capacity [48, 49]. In a set of 46 independent cultures of hMSCs, four batches of transformed MSCs were able to generate sarcoma-like tumors in immunodeficient mice [48]. The pathological features of the transformed cells include cells with spherical, cuboidal to spindly in shape, adherent, and exhibited contact-independent growth. Cytogenetic analysis showed chromosome aneuploidy and translocations with a higher level of telomerase activity compared with typical MSCs, which led to multiple solid tumors when transplanted into immune compromised host [30]. Furthermore, the transformation of hMSCs could be induced by the exogenous expression of hTERT, H-ras [50, 51]. In the course of transformation, senescence is avoided via upregulation of c-myc and repression of p16 which was accompanied with reduced mitochondrial metabolism and DNA damage repair capacity [52]. DNA hypomethylation was also indicated to occur late during stepwise MSCs transformation and was not indispensable during the process of transformation in vitro [53]. While MSC-originated tumorigenesis is mainly limited to sarcoma, it is possible that MET observed in cancer metastasis may be acquired during MSC self-transformation, leading to different epithelial source malignancy [46, 54–56]. Like the animal syngeneic MSC cancer model, it remains to be investigated whether human epithetical cancers could be rooted from hMSCs or hMSC-like intermediate forms of pre-cancer cell types via MET.

### MSCs identified in the pro-cancerous niches

Cell transformation is tightly associated with chronic inflammation or induces an inflammatory response (tumor-elicited inflammation), both supporting that chronic inflammation is an extrinsic factor for malignant cell transformation in the pre-cancerous tissue niche [57]. Probably by a similar cluster of attractors, MSCs are shown to migrate to the tumor site resembling the migration toward injured tissue. MSCs gathered from the local tissue and/or the circulating MSCs are recruited and reside in the so-called inflammatory niche (Fig. 1B) generated from different injuries, including virus infection and mechanical stress such as cut and ionizing radiation, which is documented to be a pre-cancer environment. Inflammatory cytokines such as TNF-α and IL-1β confer MSCs the ability to release high levels of CCL2, CXCL8 and CCL5, which lead to exacerbated inflammatory and pro-cancerous profiles [58]. These results demonstrate the possibility that the circulating MSCs are actively involved in creating the pro-malignant inflammatory tissue environment motivating a favorable condition for cell transformation. It remains to be examined if an unknown transition type of MSCs could be the original TICsin the pre-cancerous niches. In addition, MSCs could regulate the ecological dynamics leading to the transition of the pre-cancerous niches to the fully transformed malignant tumor microenvironment.

## MSCs contribute to pro-tumor ecological environment

**Fig. 2:**
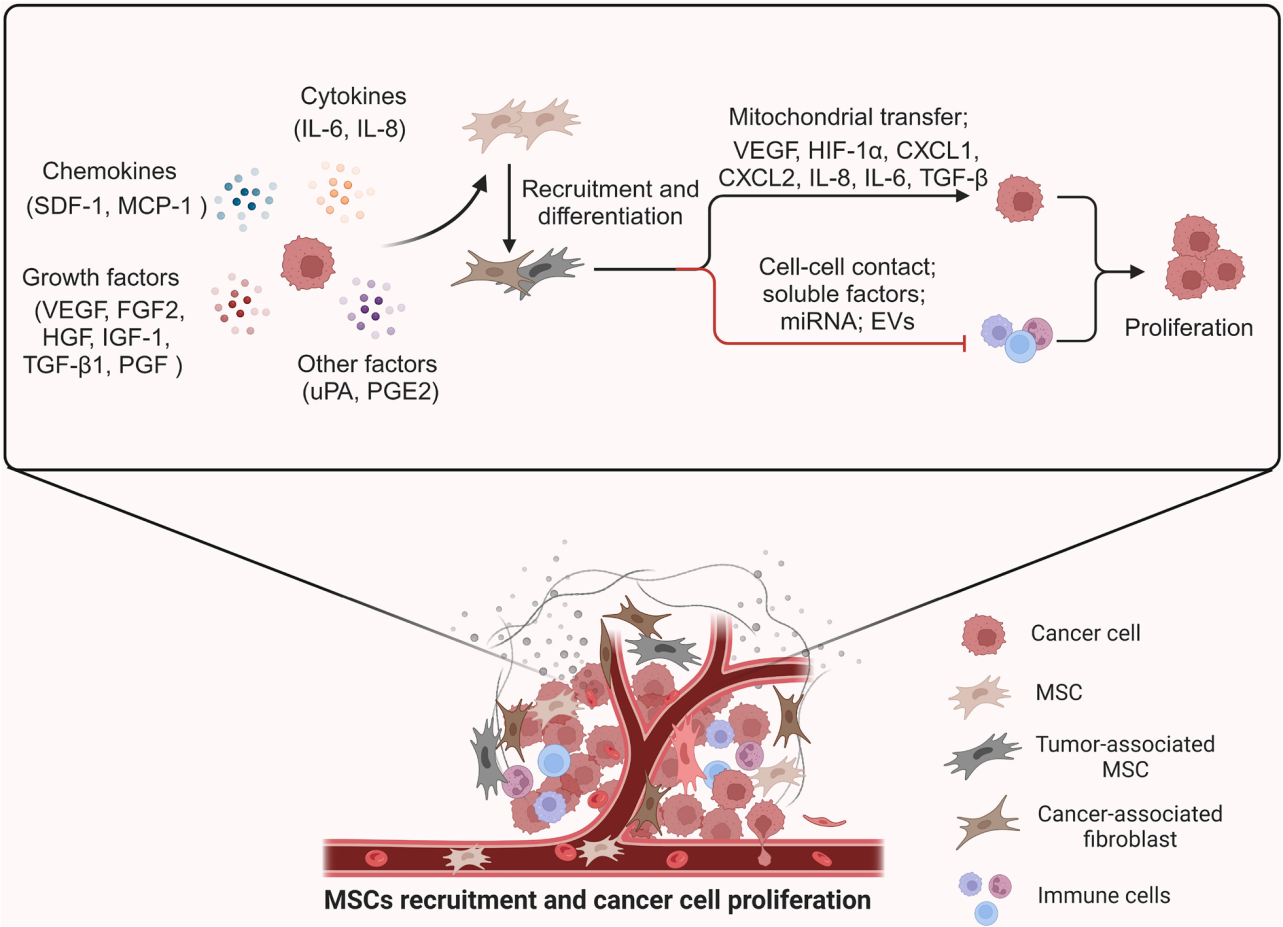
MSCs recruitment by tumor-secreted elements accelerate tumor proliferation. Tumor-secreted bioactive elements including chemokines (CXCL12, MCP-1), cytokines (IL-6, IL-8), growth factors (VEGF, FGF2, HGF, IGF-1, TGF-β1, PGF), and other factors (uPA, PGE2) favor the recruitment of MSCs into TME. In TME, MSCs can be further educated into TA-MSC and/or CAF, both promoting tumor cell proliferation via mitochondrial transfer and secreting factors including CXCL-1, CXCL-2, VEGF, TGF-β, IL-8, and IL-6. Simultaneously, immune cell response activated by inflammatory factors in TME is inhibited by recruited MSCs by cell–cell contact, soluble factors, miRNA, and EVs approaches, leading to the establishment of the immunosuppressive TME. accelerates

The ecological dynamics of TME feature the evolution of tumor cells related to the host stromal environment [59–62]. Although it is unknown how MSCs contributed to the overall ecology of TME, increasing evidence indicates that MSCs promote tumor proliferation. Tumor growth is boosted by administration of MSCs into the systemic circulation of tumor-bearing animals [63]. Orthotopic gastric tumors can be enhanced if the host mice receive transplantation of syngeneic mBM-MSCs [64]. The tumor-boosting function is also observed by co-injection of tumor cells with MSCs isolated from human head and neck carcinoma [65], gastric cancer [66], and gliomas [67], which is potentially related to the immunosuppressive function of MSCs [68]. The MSC-attracting functions of TME are generated by the multiple tumor-secreted chemokines, cytokines, and growth factors (Fig. 2) for recruiting MSCs from bone marrow or adipose tissue towards tumor xenografts. VEGF [69], FGF2 [69], PGF [70], IL-6 [71], IL-8 [72], HGF [73], SDF-1 [74], IGF-1 [75], MCP-1 [76], uPA [77], PGE2 [78], TGF-β1 [79] among others have been identified as the tumor-MSC recruiting factors. Such MSC-attracting dynamics function to recruit MSCs and educate the MSCs in the TME. Liu et al. found that MSCs mediated C26 colon cancer growth with enhanced angiogenesis if MSCs were pre-stimulated with both IFN-γ and TNF-α rather than with either IFN-γ or TNF-α alone [80]. When arriving to TME, MSCs may acutely or chronically differentiate into TA-MSCs or cancer-associated fibroblasts (CAFs) by the tumor-guided education, further assisting the malignant progression [81]. Tumor-educated MSCs can further activate and release the chemoprotective and immunomodulatory factors including CXCL1, CXCL2, and IL-8, favoring tumor progression [82] in which MSCs generated CXCL2, VEGF, TGF-β, and IL-6 can further raise tumor aggressive phenotype by boosting tumor angiogenesis [83]. MSC-mediated tumor ecologic dynamics are also related to MSC-related TME immunosuppression [84]. MSCs co-injected with inflammatory breast cancer cells can stimulate the secretion of IL-6 from macrophages which is required for the colonization of the inflammatory breast cancer [85]. Such pro-tumor ecology is further illustrated by the communication between tumor cells and stromal cells with a feed-forward loop generating a metabolic synergy for tumor energy consumption demands [86]. The tumor-educated MSCs are actively involved in the metabolic reprogramming in the TME in which the mitochondria of MSCs play a central role in driving the MSC-boosted tumor progression via an energy-transferring mechanism [87], thereby increasing cell proliferation and invasion of breast cancer and glioblastoma cells [88, 89]. Suppression of MSCs migration capacity inhibits MSC-enhanced tumor aggressive phenotype [90] in which miR-126 is shown to inhibit SDF-1α expression and diminish MSC recruitment into TME [91]. Further elucidation of the mechanistic insights and key elements required for MSC recruitment is mandatory for the invention of therapeutic approaches to reverse the proliferative tumor ecology.

## MSCs enhance tumor cell metastatic capacity and enrich metastatic tissue niche

### MSCs enhance tumor metastatic capacity

**Fig. 3:**
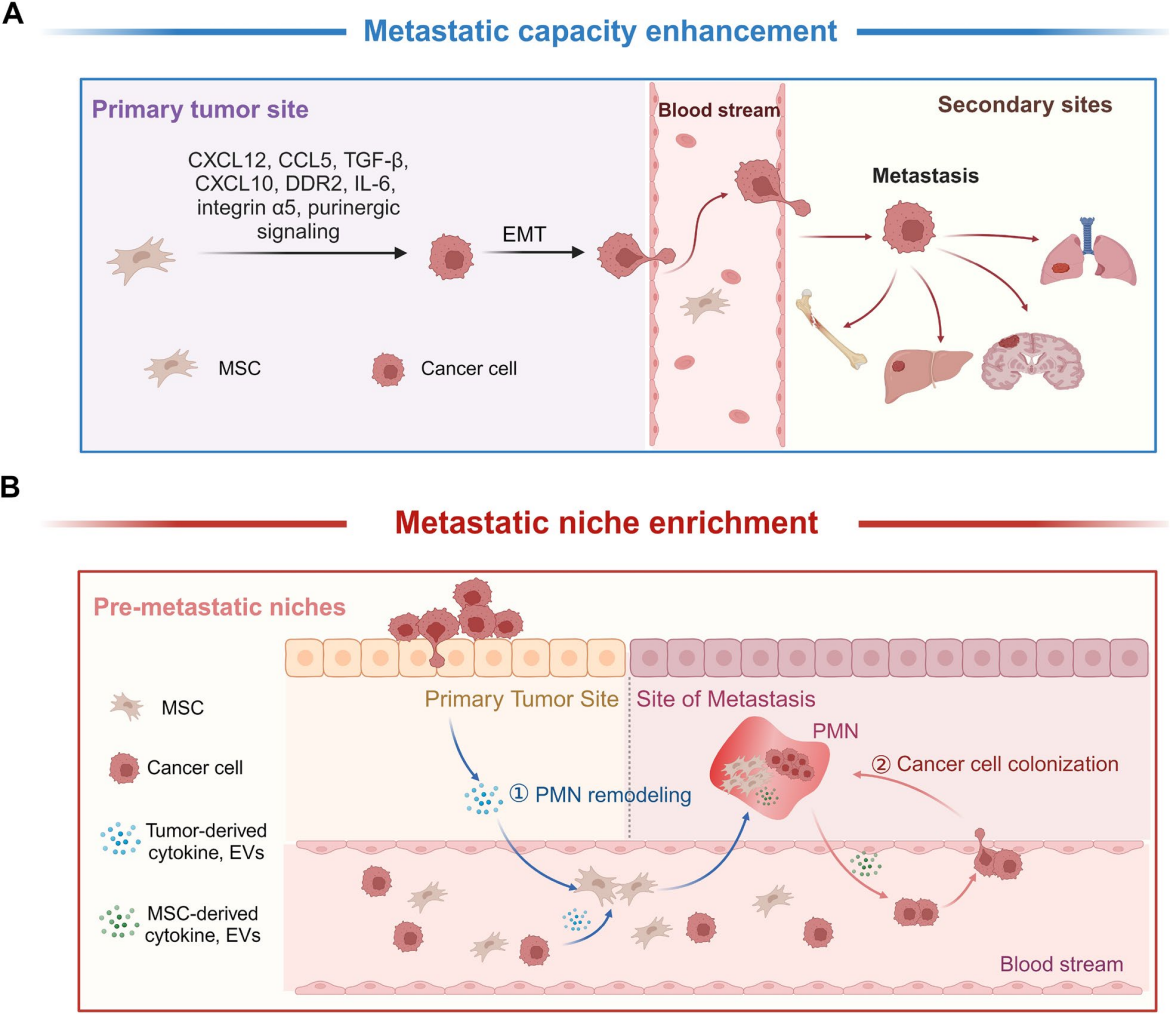
MSCs boost tumor metastasis in both seed-to-soil and soil-to-seed cascades. **A** TA-MSCs and CAFs enhance the “seed” tumor cells by releasing cytokines including CCL12, CCL5, TGF-β, CXCL10, DDR2, IL-6, integrin α5, and MSCs-derived macrovesicles, conferring tumor cells with metastatic potential mainly by EMT, which boosts local recurrence and distant metastasis. **B** Meanwhile, MSCs in TME provide the adaptive “soil” to fit into the metastatic niche for the homing tumor cells. Firstly, paracrine factors and EVs secreted by primary tumor recruit MSCs to the pre-metastatic niches (PMN) where a mature niche is developed for the anchoring of homing tumor cells which is followed by MSCs-secreted factors facilitating the cancer cell colonization at PMN

The tumor cells capable of colonization into the metastatic site can be boosted by MSC-derived cytokines (Fig. 3A). TA-MSCs and CAFs accelerate the progression of tumor cells towards a more aggressive phenotype, including invasive and pro-metastatic states [92, 93]. Myeloma cell-educated MSCs demonstrate altered differentiation and transcriptomics which fit into an efficient niche to support the survival and proliferation of the myeloma cells [94, 95]. Tumor-educated MSCs can promote epithelial-mesenchymal transition (EMT) for tumor metastasis [96, 97]. Breast cancer MDA-MB-231 cells pre-treated with mouse and human MSCs significantly increased lung metastasis [98]. CXCL16 is indicated to play a critical role in MSC-boosted tumor metastasis by facilitating MSC recruitment and conversion to CAFs that secrete CXCL12 to regulate EMT in tumor cells [99]. MSCs-derived CCL5 can raise tumor cell invasion and metastasis [100], while MSCs-released TGF-β leads to the force-dependent directional migration of invasive breast cancer cells [101]. Breast cancer metastasis is enhanced by HIF-dependent CXCL10 upregulation in MSCs [102], or by DDR2 expression in MSCs [103]. Human colorectal cancer-derived MSCs enhanced the growth and metastasis of colorectal cancer cells in vitro and in vivo via the IL-6/JAK2/STAT3 signaling pathway [104]. In addition to EMT, growth-accelerating genes including integrin α5 [105] and ionotropic purinergic signaling pathway [106] are enhanced in MSCs-mediated tumor cell metastatic potential.

### MSCs enrich metastatic niche

The pre-metastatic niche (PMN) forms a permissive environment facilitating the metastatic cell implantation and providing a context for the selection of cells capable of surviving and thriving in the new tissue environment [107]. PMN is established by modulation of the local ecological conditions including cell repopulation and nutrient adjustment and by preconditioning BMSCs that migrate and prepare the parenchyma for cancer cell colonization [108]. The MSCs in the PMN provide the tissue-specific pro-transformation status of BRCA1/2-mutant mediated breast and ovary cancers [60]. Mesenchymal gene expression has been observed in tumor bone metastases, indicating that mesenchymal signals from the primary tumor stroma may promote distant metastasis [109]. In this regard, the MSCs already educated by the primary tumor cells may travel in the system and function to sustain and promote the PMN [82, 110]. Paracrine factors and EVs secreted by the primary tumor are involved in recruiting the BMSCs to the second site to develop a mature niche for tumor cell metastasis [111]. In prostate cancer bone marrow metastasis, the PMN is assumed to be established via exosome pyruvate kinase M2 to promote metastasis [112]. Exosomes secreted by prostate cancer cells enhance the activity of matrix metalloproteinase in the PMN leading to extracellular matrix remodeling required for the recruitment of bone marrow cells to the PMN [113]. BMSCs play an essential role in bone homeostasis; failures in their functionality can cause osteolysis [114] which favors PMN [115]﻿ to home circulating tumor cells [116]. A CXCL12-enriched bone marrow PMN is identified to enhance the clonal seeding of triple-negative breast cancer metastasis [117]. BMSCs-secreted IL-6, IL-8, LIF, GM-CSF, ICAM-1 and MMP-3 are involved in bone remodeling which further supports the metastatic cell colonization [111]. Together, these findings indicate that MSCs and circulating tumor cells and their released immunosuppressive cytokines, coordinatively create PMN for tumor metastasis (Fig. 3B).

## MSCs contribute to immunosuppressive TME

### MSCs target immune cells

**Fig. 4:**
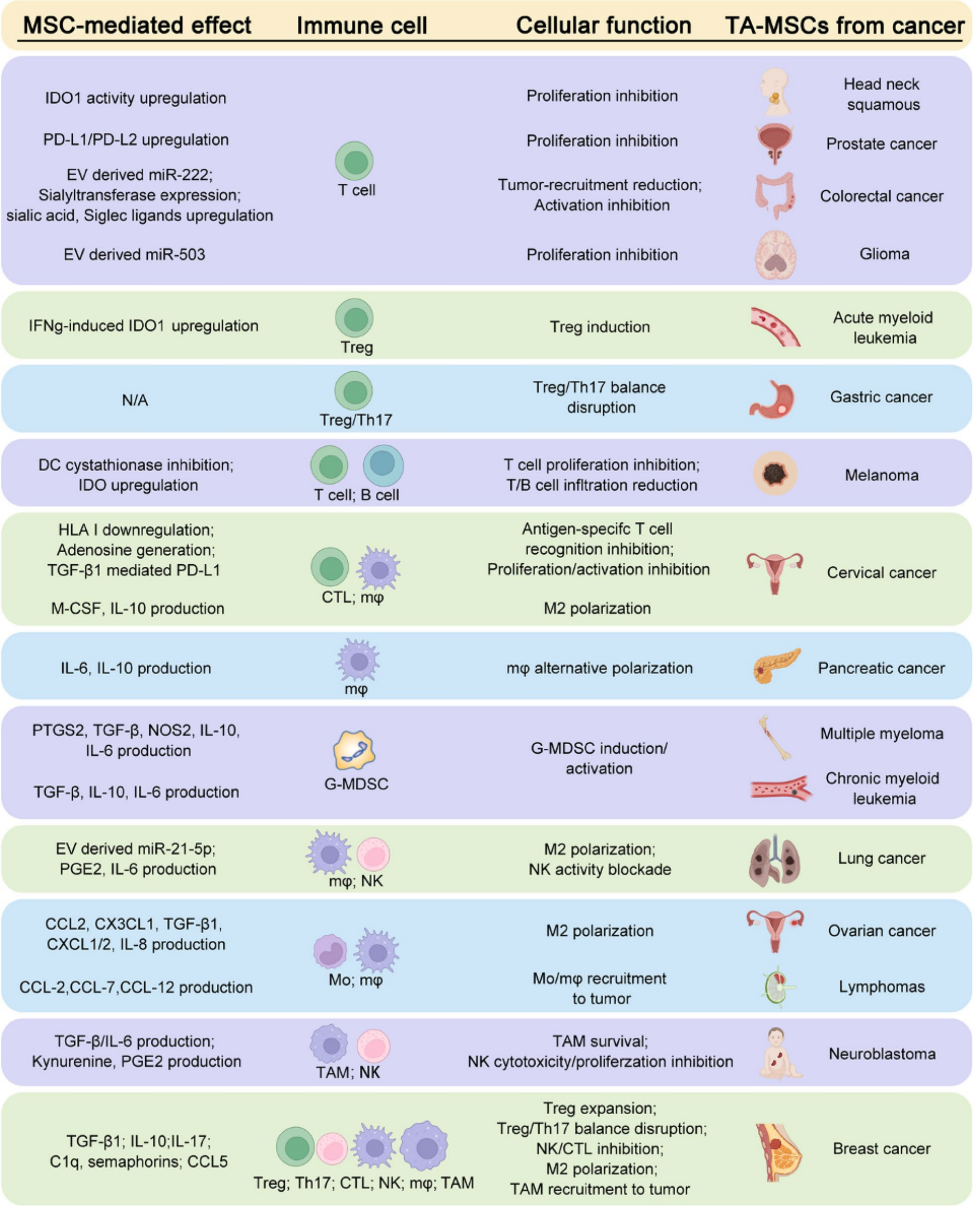
MSCs regulating TME immune cells. **A** Schematic is generated with the data of MSCs-mediated bioactive signals that are demonstrated to regulate specific clusters of immune cells and the consequences. **B** Experimental and clinical information were collected regarding the therapeutic approaches targeting the immune cells in TME of the cancer located in the indicated organs

In addition to the well-defined immunosuppressive cells including Tregs, M2 macrophages, and MDSCs [118], the immunosuppressive tumor microenvironment is significantly influenced by TA-MSCs or CAFs that release molecules to inhibit immune surveillance or induce the EMT of tumor cells, resulting in tumor migration and invasion [99]. MSC-derived cytokines and growth factors promote an immunosuppressive environment that leads to inhibition of the adaptive immune system [63]. This immunosuppressive function of MSCs can be further enhanced in the TME through re-education, which could sustain a high level of various inflammatory factors and further recruit circulating MSCs and immunosuppressive cells. About 1–5% of MSCs were found in endometrial cancer and the population of MSCs in tumor tissues was correlated with the progressive status and expression level of the programmed death ligands PD-L1 and PD-L2 [119]. The MSCs residing in the TME can stimulate tumor growth by promoting immunosuppression [120], which is supported by many studies indicating that all immune cells, including T cells, B cells, macrophages, NK cells, and DC, can be targeted by MSCs leading to the immunosuppressive tumor status (Fig. 4, Table 1). In the TME of breast and prostate cancers, MSCs can defend tumor cells by upregulating Treg cells, promoting M2 polarization, and downregulating NK cells and cytotoxic T lymphocyte (CTL) [121, 122]. The immunosuppressive effect of MSCs is predominantly elicited by IFN-γ and the concomitant presence of any of TNFα, IL-1α, or IL-1β, which induce a high expression of inducible nitric oxide synthase by MSCs, inhibiting T cell response [123]. Furthermore, TA-MSCs can inhibit DCs' ability to promote the expansion of naïve CD4^+^ and CD8^+^ T cells, the secretion of IFN-γ, and the cytotoxic functions of T cells on tumor cells through an IL-10-STAT3 dependent pathway [124]. Immunosuppressive TA-MSCs are indicated to promote M2 polarization limiting the phagocytotic attack on tumor cells and favoring tumor progression [82]. TA-MSCs are also involved in educating macrophages by manipulating metabolic programs in differentially polarized macrophages [125]. Moreover, transferring MSCs-derived mitochondria to T cells caused Treg generation restricting the inflammatory response [126]. These results demonstrate that different immune functions are activated in the TME by targeting varied immune cells or subtypes of immune cell populations. Intriguingly, low-dose radiation-treated MSCs have been shown to reduce immune suppression, favoring the anti-tumor action of the immune system in mouse glioblastoma [127]. Together, TME-recruited MSCs generally create a pro-tumor immunosuppressive environment, and targeting TA-MSCs is an attractive approach to raising tumor response to ICB. In addition, TA-MSCs are specifically expanded by metastatic tumor cells and are more powerful than MSCs not educated by cancer cells in promoting tumor progression and dissemination accompanied by immune suppression [85, 128]. Further elucidation of the secretomics from circulating MSCs versus resident MSCs of normal and tumor tissues may provide more insights into MSCs-regulated tumor immunosuppression.

**Table 1:** MSCs interaction with immune cells in TME

Target immune cell	Cancer type	Function	Mechanism involved	Refs
T cell	Head neck squamous	Proliferation inhibition	IDO dependent	[129]
T cell	Prostate cancer	Proliferation inhibition	PD-L1/PD-L2 upregulation	[122]
T cell	Melanoma	Proliferation inhibition	DC cysteine export via IL-10-STAT3	[124]
T cell	Colorectal cancer	Tumor-recruitment reduction	MSC-EV derived miR-222	[130]
T cell	Colorectal cancer	Activation inhibition	Sialyltransferase expression, sialic acid, and Siglec ligands	[131]
T cell	Glioma	Proliferation inhibition	MSC-EV derived miR-503	[132]
T cell, B cell	Melanoma and lymphoma	Tumor-infiltrating reduction	IDO dependent	[133]
CTL	Cervical cancer	Antigen-specific T cell recognition inhibition	HLA class I downregulation	[134]
CTL	Cervical cancer	Proliferation/activation inhibition	Adenosine dependent	[135]
CTL	Cervical cancer	Proliferation/activation inhibition	TGF-β1 mediated PD-L1	[136]
Treg	Acute myeloid leukemia	Treg induction	IFNγ induced IDO1	[137]
Treg	Breast cancer	Expansion	IL-10 and TGF-β dependent	[138]
Treg/Th17	Gastric cancer	Treg/Th17 balance disruption	N/A	[139]
Treg/Th17	Breast cancer	Treg/Th17 balance disruption	TGFβ and IL-17 dependent	[140]
Treg/NK/CTL	Breast cancer	Treg expansion; NK/CTL inhibition	TGF-β1 dependent	[121]
NK cell	Lung cancer	Activity blockade	PGE2 and IL-6 dependent	[141]
NK cell	Neuroblastoma	Cytotoxicity/proliferation inhibition	Kynurenine and PGE2 dependent	[142]
Macrophage	Breast cancer	M2 polarization	MSC-derived exosomes containing TGF-β, C1q, semaphorins	[143]
Macrophage	Pancreatic cancer	Alternative polarization	IL-6 and IL-10 dependent	[144]
Macrophage	Lung cancer	M2 polarization	MSC-EV derived miR-21-5p	[145]
Macrophage	Cervical cancer	M2 polarization	M-CSF and IL-10 dependent	[146]
TAM	Neuroblastoma	Survival	TGF-β/IL-6 pathway	[147]
TAM	Breast cancer	Tumor recruitment	CCL5/CCR5/CSF1/CSF1R loop	[148]
Monocyte	Follicular lymphoma	Recruitment and TAM differentiation	CCL-2 dependent	[149]
Monocyte	Ovarian cancer	M2 macrophage polarization	CXCL1/2 and IL-8 dependent	[82]
Monocyte, macrophage	Ovarian cancer	Ccr2^+^ monocyte/TAM recruitment	CCL-2, CX3CL1, TGF-β1dependent	[150]
Monocyte, macrophage	Lymphomas	Tumor recruitment	CCL-2, CCL-7, CCL-12 dependent	[151]
G-MDSC	Chronic myeloid leukemia	Activation	TGFβ, IL-10, IL-6 dependent	[152]
G-MDSC	Multiple myeloma	Induction	PTGS2, TGFβ, NOS2, IL-10, IL-6 dependent	[153]

### Immunosuppressive MSC-EVs

MSC-EVs are nano-sized double-membraned vesicles acting as paracrine effectors of MSCs. The cargo of MSC-EVs contains a diverse range of bioactive molecules, such as proteins, miRNAs, and lipids with great potential in immune modulation, including targeting innate and adaptive immune cells like macrophages, granulocytes, mast cells, NK cells, DCs and lymphocytes [154, 155]. MSC-EVs are actively involved in cell–cell communication within the TME. Exosomes (EXOs) being the major type of EVs, play a critical role in regulating tumor proliferation, aggressive behavior of metastatic tumors, and chemoresistance and thus the potential therapeutic targets [156–160]. Increasing secretomics analysis has revealed a wide scale of immune regulating molecules in the cargo of MSC-EVs, including non-coding RNAs, miRNAs, long ncRNAs, transcription factors, and nucleic acids. EXOs secreted by tumor-educated MSCs can enhance breast cancer progression by inducing MDSC differentiation into immunosuppressive M2 macrophages. Inquiringly, MSC-EXOs but not EXOs from tumor cells contain TGF-β, C1q, and semaphorins, increasing the myeloid tolerogenic activity with PD-L1 overexpression in immature myelomonocytic precursors and committed CD206^+^ macrophages [143]. This result indicates that MSC-EXOs can promote MDSCs differentiation into protumor M2 macrophages leading to tumor immune evasion. Another study has revealed that MSC-EVs are capable of transporting miR-222 targeting ATF3, leading to AKT1 transcriptional suppression, and consequently enhancing malignant aggressiveness and immune escape [130]. Elevated delivery of miR-21-5p by MSC-EVs following hypoxia pre-challenge fosters lung cancer development through apoptosis reduction and facilitation of macrophage M2 polarization [145]. Hyaluronic acid (HA) secreted from TA-MSC-EVs is associated with GBM aggressiveness [159, 160]. Bioengineered MSC-EXOs are currently in pre-clinical and clinical testing stages [161, 162]. The development of more effective MSC-EV targets relies on further characteristics of the in vivo MSCs and the high heterogeneity including the great scale of various contents carried by the MSC-EVs from tumor-educated and non-educated MSCs. These studies will provide critical information to invent new therapeutic targets for enhancing cancer control by ICB.

## Fusion of MSCs with other cells

It remains unclear why MSCs are fusing with other somatic cells under physiological and pathological conditions, although such fusion is an infrequent event. In vitro settings, fusion between MSCs and human breast epithelial MCF10A cells can be boosted by TNF-α mediated apoptotic response [163], whereas, EMT and malignant transformation are initiated from the fusion of MSCs with gastric epithelial cells [164]. The fusion of hMSCs delivered to the damaged murine heart is detected in the target organ and surrounding organ systems. The migration of hMSCs fusion products to distal organs is primarily located close to the vasculature, indicating that cell fusion requiring cell mobility is linked with the blood vessel velocity [165]. Experimental evidence also shows that the occurrence of cell fusion mainly depends on the density of the cells, the cell ratio of the parental populations, the components of the medium, and culture conditions [166]. In contrast, MSC-tumor cell fusion has been extensively studied. Spontaneous hybrid cells are identified in hMSCs co-cultured with an array of cancer cells. The hybrids seem to acquire a mixed property of functions inherited from both parental cell types (MSCs and cancer cells), including the expression of specific markers of the two cells, increased proliferation, migration capacity, and stemness [167]. The engulfment of MSCs by MDA-MB-231 breast cancer cells can enhance breast cancer cell metastatic potential, resulting in hybrid cells with mesenchymal-like, invasion, and stem cell traits [168]. In vivo tests further demonstrate that such MSC-cancer cell hybrids have elevated tumorigenicity and metastatic potential with enhanced tumor heterogeneity in breast cancer [168–171]. Interestingly, microarray-based mRNA profiling of the hybrids defined a cluster of genes for EMT and metastasis-associated S100A4 and ZEB1, with decreased expression of CK-18 [168, 171]. The MSC-engulfed breast cancer MDA-MB-231 cells demonstrated enhanced EMT and invasive potential [168]. Although the precise mechanisms underlying MSC-tumor cell fusion remain to be elucidated, hypoxic condition-induced cell apoptosis could be a prerequisite for the fusion [172], thus the phenomenon of MSC-tumor cell fusion could indicate a form of adaptive prosurvival function of tumor cells. The physiological and pathological fusion of MSC-normal and MSC-tumor cells require further investigation.

## Targeting MSCs in cancer radiotherapy

A dual function of normal tissue radioprotection and inhibiting tumor cells by MSCs is suggested. MSCs are shown to repair radiation-induced normal tissue injuries [173] whereas targeting TA-MSCs is an attractive approach for the regulation of immune status in TME [120]. The idea of MSCs-mediated radiation damage repair is encouraged by the finding that radiation can activate MSCs metabolism, thus enhancing MSCs functional activity [174]. Preclinical studies have indeed demonstrated the potential of MSCs being recruited to the radiation-induced lesion sites, repairing tissue damage and supporting the regeneration of functional tissues [175], especially in the restoration of the radiation-injured intestine [176], lung [177], and skin [178] as well as to mitigate premature ovarian failure [179]. Following the aforementioned tumor-promoting function of TA-MSCs, it is thus to be carefully balanced on an MSCs-targeted approach in cancer radiotherapy. It is already demonstrated that in TME, irradiation-induced cytokines secreted by 4T1 cells, including TGF-β1, VEGF, and PDGF-BB, can facilitate MSCs chemotaxis towards the tumor site [180]. Furthermore, CCL2 can act as a factor in the IR-induced tropism of MSCs to provide pro-tumor gliomas TME [181]. Conversely, irradiated MSC-EXOs enhanced tumor radiation response, improving the control of melanoma cell growth and metastasis [182]. Similarly, MSCs could potentially promote the effect of radiotherapy in colorectal cancer by secreting TNF-α and IFN-γ [183]. Based on these reports, currently, the exact activities of the tissue-resident MSCs in TME under therapeutic irradiation, especially the TA-MSCs in recurrent and metastatic lesions after radiotherapy, need to be further elucidated. The heterodetic MSCs sub-populations, dynamic communication, as well as potential MSC-tumor or other stromal cell fusion in the irradiated TME, are currently unknown. It is thus expected that a specific subtype of TA-MSCs or evolution of TA-MSC could drive tumor aggressive growth or metastasis.

## Conclusions and perspectives

This review describes the widescale functions of MSCs from different resources including their potential self-transformation and acquiring tumor-promoting functions, serving as TICs. A fundamental question that remains unanswered is whether the original malignant epithelial cell(s) arise through MET of MSCs derived from tissue residence and/or circulation. In an age of multi/single genome omics and spatial transcriptomics, reevaluating the fundamental traits and tracking of cancer-initiating MSCs could represent a significant breakthrough in our comprehension of cancer cell origin and potential prevention [184, 185]. Increasing evidence has revealed an important role in tumor immune regulation especially the tumor-infiltrated immune cells targeted by both circulating and tissue-resident MSCs. In this regard, further characterization of the heterogeneity of MSCs in TME may help to clarify the clusters responsible for carcinogenesis and progression [186]. These insights will also be informative for the application of MSCs and MSC-EVs in the fields of regenerative medicine using MSCs from different tissue resources, although current clinical evidence doesn't strongly suggest MSC-originated malignancy. Lastly, to enhance the effectiveness of cancer immunotherapy, it is imperative to gain a comprehensive understanding of the dynamic interactions between MSCs and various immune cells within the TME.

## Data Availability

No datasets were generated or analysed during the current study.

## Abbreviations

ATF3: Activating transcription factor 3
BMSCs: Bone marrow-derived mesenchymal stromal cells
CAFs: Cancer-associated fibroblasts
CCL: Chemokine (C–C motif) ligand
CXCL: Chemokine (C-X-C motif) ligand
CTL: Cytotoxic T lymphocyte
CK-18: Keratin 18
DCs: Dendritic cells
DDR2: Discoidin domain receptor 2
ECM: Extracellular matrix
EMT: Epithelial-mesenchymal transition
EVs: Extracellular vesicles
EXOs: Exosomes
ELMO1: Engulfment and cell motility protein 1
FGF2: Fibroblast growth factor 2
FTEM: Far tumor microenvironment
GM-CSF: Granulocyte–macrophage colony-stimulating factor
HGF: Hepatocyte-growth factor
hMSCs: Human mesenchymal stromal cells
HIF: Hypoxia-inducible factor
IGF-1: Insulin-like growth factor-1
ICAM-1: Intercellular adhesion molecule 1
IL: Interleukin
IFN-γ: Interferon-γ
ITME: Irradiated tumor microenvironment
LIF: Leukemia inhibitory factor
NKs: Natural killer cells
MSCs: Mesenchymal stromal cells, also termed as mesenchymal stem cells, mesenchymal stem-like cells
MCP-1: Monocyte chemotactic protein-1
MDSCs: Myeloid-derived suppressor cells
MET: Mesenchymal-epithelial transition
mMSCs: Mouse mesenchymal stromal cells
NO: Nitric oxide
MSR1: Macrophage scavenger receptor
PDGF-BB: Platelet-derived growth factor BB
PGF: Placental growth factor
PGE2: Prostaglandin E2
PCa: Prostate cancer
PMN: Pre-metastatic niche
PD-L1: Programmed death-ligand 1
SCs: Stem cells
SDF-1: Stromal-cell derived factor
S100A4: S100 calcium binding protein A4
SIRPB1: Signal regulatory protein beta 1
TME: Tumor microenvironment
TA-MSCs: Tumor-associated mesenchymal stromal cells
TGF-β: Transforming growth factor-β
TICs: Tumor-initiating cells, also termed cancer stem cells
Tregs: Regulatory T cells
TNF-α: Tumor necrosis factor α
uPA: Urokinase plasminogen activator
VEGF: Vascular endothelial growth factor
ZEB1: Zinc finger E-box binding homeobox 1
